# Annotation Query (AnnoQ): an integrated and interactive platform for large-scale genetic variant annotation

**DOI:** 10.1093/nar/gkac418

**Published:** 2022-05-30

**Authors:** Zhu Liu, Tremayne Mushayahama, Bryan Queme, Dustin Ebert, Anushya Muruganujan, Caitlin Mills, Paul D Thomas, Huaiyu Mi

**Affiliations:** Division of Bioinformatics, Department of Population and Public Health Sciences, Keck School of Medicine, University of Southern California, Los Angeles, CA 90089, USA; Division of Bioinformatics, Department of Population and Public Health Sciences, Keck School of Medicine, University of Southern California, Los Angeles, CA 90089, USA; Division of Bioinformatics, Department of Population and Public Health Sciences, Keck School of Medicine, University of Southern California, Los Angeles, CA 90089, USA; Division of Bioinformatics, Department of Population and Public Health Sciences, Keck School of Medicine, University of Southern California, Los Angeles, CA 90089, USA; Division of Bioinformatics, Department of Population and Public Health Sciences, Keck School of Medicine, University of Southern California, Los Angeles, CA 90089, USA; Division of Bioinformatics, Department of Population and Public Health Sciences, Keck School of Medicine, University of Southern California, Los Angeles, CA 90089, USA; Division of Bioinformatics, Department of Population and Public Health Sciences, Keck School of Medicine, University of Southern California, Los Angeles, CA 90089, USA; Division of Bioinformatics, Department of Population and Public Health Sciences, Keck School of Medicine, University of Southern California, Los Angeles, CA 90089, USA

## Abstract

The Annotation Query (AnnoQ) (http://annoq.org/) is designed to provide comprehensive and up-to-date functional annotations for human genetic variants. The system is supported by an annotation database with ∼39 million human variants from the Haplotype Reference Consortium (HRC) pre-annotated with sequence feature annotations by WGSA and functional annotations to Gene Ontology (GO) and pathways in PANTHER. The database operates on an optimized Elasticsearch framework to support real-time complex searches. This implementation enables users to annotate data with the most up-to-date functional annotations via simple queries instead of setting up individual tools. A web interface allows users to interactively browse the annotations, annotate variants and search variant data. Its easy-to-use interface and search capabilities are well-suited for scientists with fewer bioinformatics skills such as bench scientists and statisticians. AnnoQ also has an API for users to access and annotate the data programmatically. Packages for programming languages, such as the R package, are available for users to embed the annotation queries in their scripts. AnnoQ serves researchers with a wide range of backgrounds and research interests as an integrated annotation platform.

## Graphical Abstract

**Graphical Abstract:**
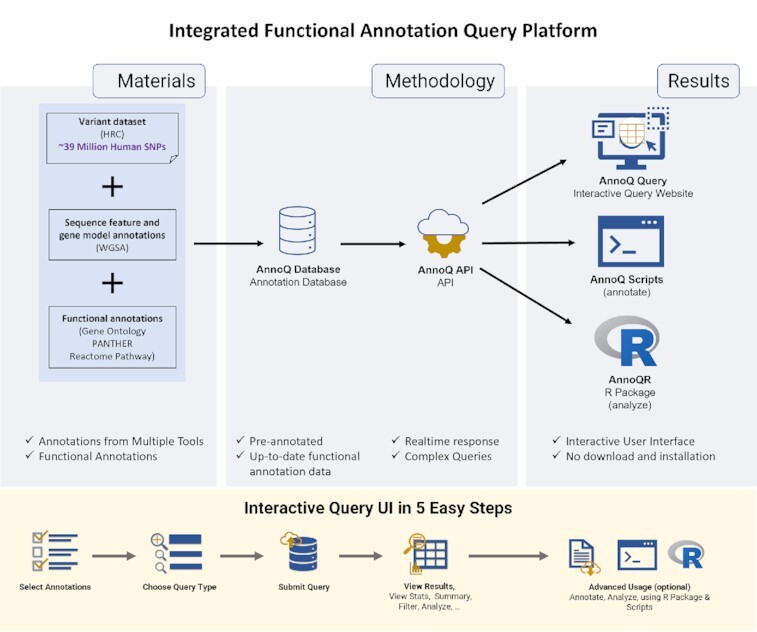
Here we present an integrated functional annotation platform for large-scale genetic variant annotation called Annotation Query (AnnoQ) (http://annoq.org). The core of the system is the AnnoQ Database, a pre-annotated variants database optimized for search and retrieval using Elasticsearch technologies. Annotation queries can be performed through an interactive web interface, an API or a programming package (R package).

## INTRODUCTION

Genetic variants have been shown to determine the diversity of human traits and phenotypes and be associated with risks for many human diseases (1–4). With the improvement of sequencing technology, a growing number of human variants have been identified. Large-scale association studies have been conducted aiming to find the links between variants and diseases. The results will help us in understanding the mechanism of the diseases, designing diagnostics and therapeutics, and following up on the prognosis. One key element of these studies is to interpret the results, i.e. to understand the underlying functional relevance of the variants. Annotation of genetic variants becomes crucial for accurately understanding and interpreting the data.

During the past decade, several tools and resources have been developed to provide gene-model or sequence feature annotations, such as ANNOVAR (5), SnpEff (6) and VEP (7). In addition, resources and databases have been developed to focus on the annotations in specific areas, such as disease (e.g. COSMIC (8), ClinVar (9)), allele frequencies (e.g. 1000 genome (10), gnomAD (11)), epigenomics (e.g. ENCODE (12), FANDOM (13)) and functional mutations (e.g. SIFT (14), REVEL (15)). Nearly all of these resources require some computational or bioinformatics skills to use them. There is no standard way to set them up because each of them has its own system requirements and supports different versions of data (e.g. genome builds, gene or protein identifiers, etc.). Integrated analyses of variants are often handled by core facilities and teams with such specialties. As a result, they are not routinely used by researchers without informatics support, such as many bench scientists and statisticians, especially when they only need to annotate a few variants at a time.

Functional annotation data, such as pathways or biological functions and processes, are essential in interpreting large-scale association data. However, these data are missing in most existing tools and resources for variant annotation. As a result, users often have to rely on additional annotation tools to get them.

An integrated and centralized system that can provide easy-to-access annotations from various variant annotation tools and resources, combined with the most updated functional annotation data will greatly help the research community. Another advantage of an integrated functional annotation query platform is that the users can query annotations from multiple tools and resources simultaneously. It is known that tools, such as gene model-based software, may employ similar annotation methods but differ in modeling details, which may result in slightly different annotations. These kinds of differences will have a substantial effect on annotation results (16). The simultaneous query of multiple annotations allows users to have a more comprehensive picture of the data, make better decisions to select the annotation data for analysis, and interpret the results more accurately.

To address all these needs, we developed an integrated functional annotation platform for large-scale genetic variant annotation called Annotation Query (AnnoQ) (http://annoq.org). AnnoQ is a powerful suite of software tools that provide resources for pre-annotated human variants information queries. The core of the system is the **AnnoQ Database**, a pre-annotated variants database optimized for search and retrieval using Elasticsearch technologies (see Elasticsearch Guide at https://www.elastic.co/guide/en/elasticsearch/reference/master/index.html). The **AnnoQ API** is intermediary software that acts as a communication tool to help simplify access, interaction, and extraction of data from the database. Accessing and interacting with the data using a graphical user interface is done through an **AnnoQ Query UI** website. The AnnoQ Query UI’s easy-to-use user interface and its search capabilities provide easy access for scientists with fewer bioinformatics skills, including bench scientists and statisticians. Programming packages, such as **AnnoQR** (R package), are available to assist in fine-tuning queries and aggregating information to serve researchers with advanced computer and bioinformatics skills.

## MATERIALS AND METHODS

**Figure 1.**
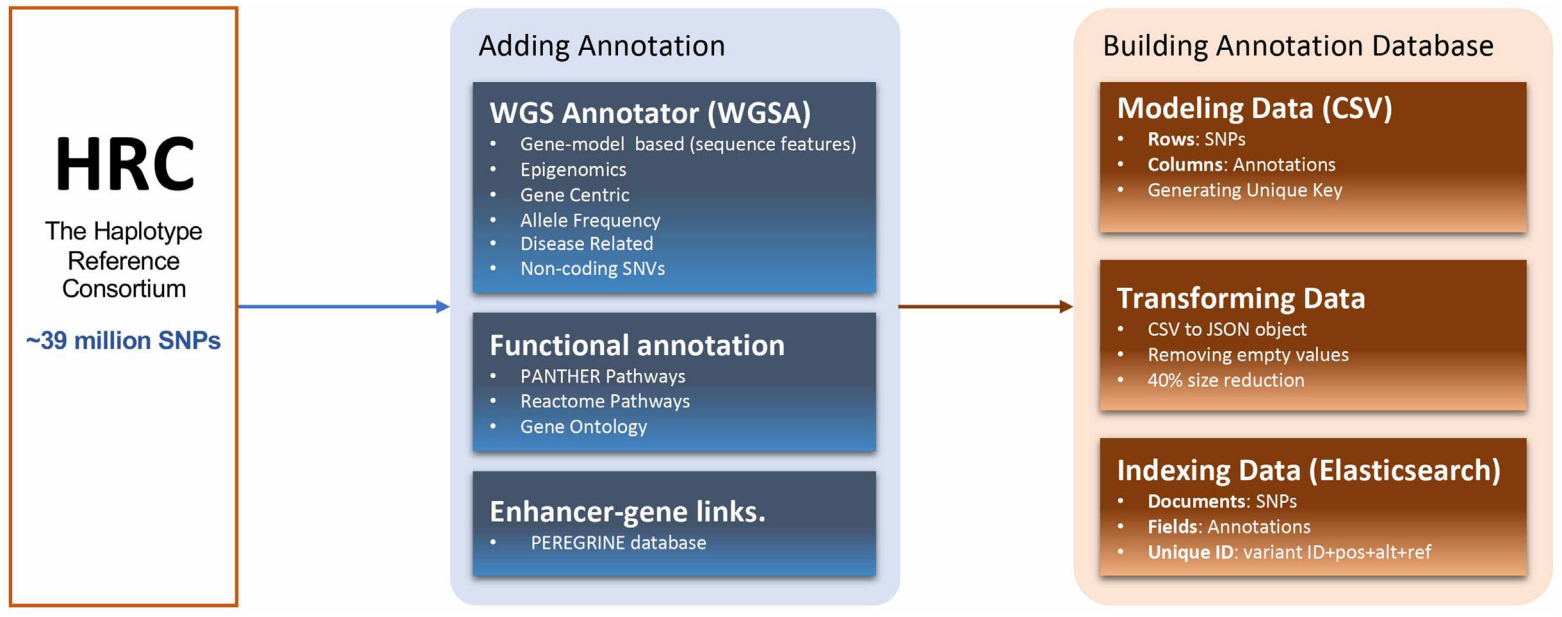
Overview of AnnoQ annotation data building process.

The AnnoQ building process is summarized in Figure 1.

### Gathering genetic variant data

The human genetic variant dataset was obtained from The Haplotype Reference Consortium (HRC) (17). It consists of about 39.24 million SNPs (GRCh37/hg19). The dataset will be updated annually if there is a new release from HRC.

### Adding annotation of variants

All variants were annotated with the following three types of data (Figure 1).

1. *Annotations from WGS Annotator (WGSA)*. WGSA (18) provides gene-model-based annotations from ANNOVAR (5), SnpEff (6), and VEP (7), as well as annotations for functional prediction scores, and conservation scores, allele frequencies, and disease-related scores. The main reasons that we choose WGSA are because it packages all the necessary tools for variant annotation and is actively maintained. It is up-to-date and easy to set up. The tool was downloaded and installed locally according to the instructions (18). All variants from HRC were annotated using the WGSA pipeline.
2. *Functional annotation*. This effort leverages the PANTHER Classification System (19,20), which has integrated Gene Ontology (GO) (21), Reactome (22), and PANTHER pathway annotation data (23,24) for genome-wide gene function analysis. These are two types of annotations using this process. First, variants were annotated based on direct mapping to nearby gene(s). We first identify genes where the variant is located in (marked as 0 flanking base pairs) or within 10k or 20k base pairs flanking regions. Then the annotations to the gene(s) are retrieved from PANTHER API (http://pantherdb.org/services/openAPISpec.jsp) (19) and assigned to the variants. Second, variants were annotated to the genes by ANNOVAR, SnpEff and VEP. Functions annotated to these genes were retrieved from PANTHER and assigned to the variants. Although ANNOVAR, SnpEff and VEP sometimes provide GO annotations, they are not complete and usually outdated. The functions annotated here are more comprehensive and up-to-date.
3. *Enhancer-gene links*. Variants that are located in enhancer regions were identified using the PEREGRINE database (25). They are given functional annotations based on the target genes linked to the enhancers predicted in PEREGRINE.

The annotations will be updated quarterly.

### Comparison of different gene model annotation tools

Gene annotations from ANNOVAR, SnpEff and VEP were gathered from the AnnoQ annotation database. For each variant, a combined list of Gene IDs was generated by pooling all the genes which were annotated by any one of the three tools. Then the list of Gene IDs from each tool was compared to the above-combined list. Since the combined list is a combination of lists from all three tools, the list from the individual tool is either the same as or a subset of the combined list. If the lists of three individual tools are all the same as the combined list, the variant is labeled as ‘3 tools agree’. This means all three tools provide the same annotations. If lists from two tools match the combined list, it is labeled as ‘2 tools agree’. This means that the list of the 3rd tool is a subset of those in the other two tools. If only one tool's list matches the combined list, it is labeled as ‘1 tool agrees’. It also means that the other two tools’ lists are subsets of the combined list. The category ‘no tool agrees’ indicates no list matches with the combined list, meaning at least one tool's list is partially or not overlapping with those of the other tool(s). In addition, we kept track of which tools had the highest matching rates. This analysis was done post-annotation and the results do not appear in the AnnoQ output. However, we may consider adding this feature in the future. In the meantime, the code for the comparative analysis is available in this project's GitHub repository.

### Building annotation database

The initial variant annotation files were generated as CSV files. Each row is a unique variant and each column is an annotation type. The data were then transformed into JSON format to support efficient search and to reduce the file size by eliminating empty values, and indexed into the Elasticsearch database (Figure 1). Elasticsearch, which is open source, serves both as a data store and text search engine. The detailed procedure can be found in the GitHub repository at https://github.com/USCbiostats/annoq-database.

### Building application programming interface (API)

Elasticsearch itself already supports basic search functions. In order to make the search more extensible, an application programming interface (AnnoQ API) was built by wrapping the original Elasticsearch with additional search functions using Python Flask. The implementation also solves some security issues brought by Elasticsearch. Most of the queries will be translated into the combination of 3 types of basic search: ID search, chromosome position range search, and keyword search in full-text. For gene search, the input gene ID is first converted into a position range and then undergoes a position range search. All annotation fields are stored as a string or numeric data type. For strings, users may perform a full-text search, e.g. a gene name, a GO term, or a pathway name. Multiple clauses can be combined into a complex query. Data have been spliced into partitions based on chromosome numbers to accelerate queries. The wrapping layer also supports configuration files (see below) parsing and other website-related functions. It is easy to extend the entire toolset by adding new functions into this layer without changing the Elasticsearch index. An additional retrieval function was implemented for large-scale data exporting and is especially useful for queries using a VCF file. More details about the AnnoQ API development can be found at https://github.com/USCbiostats/annoq-api.

## RESULTS

### Overall architecture

**Figure 2.**
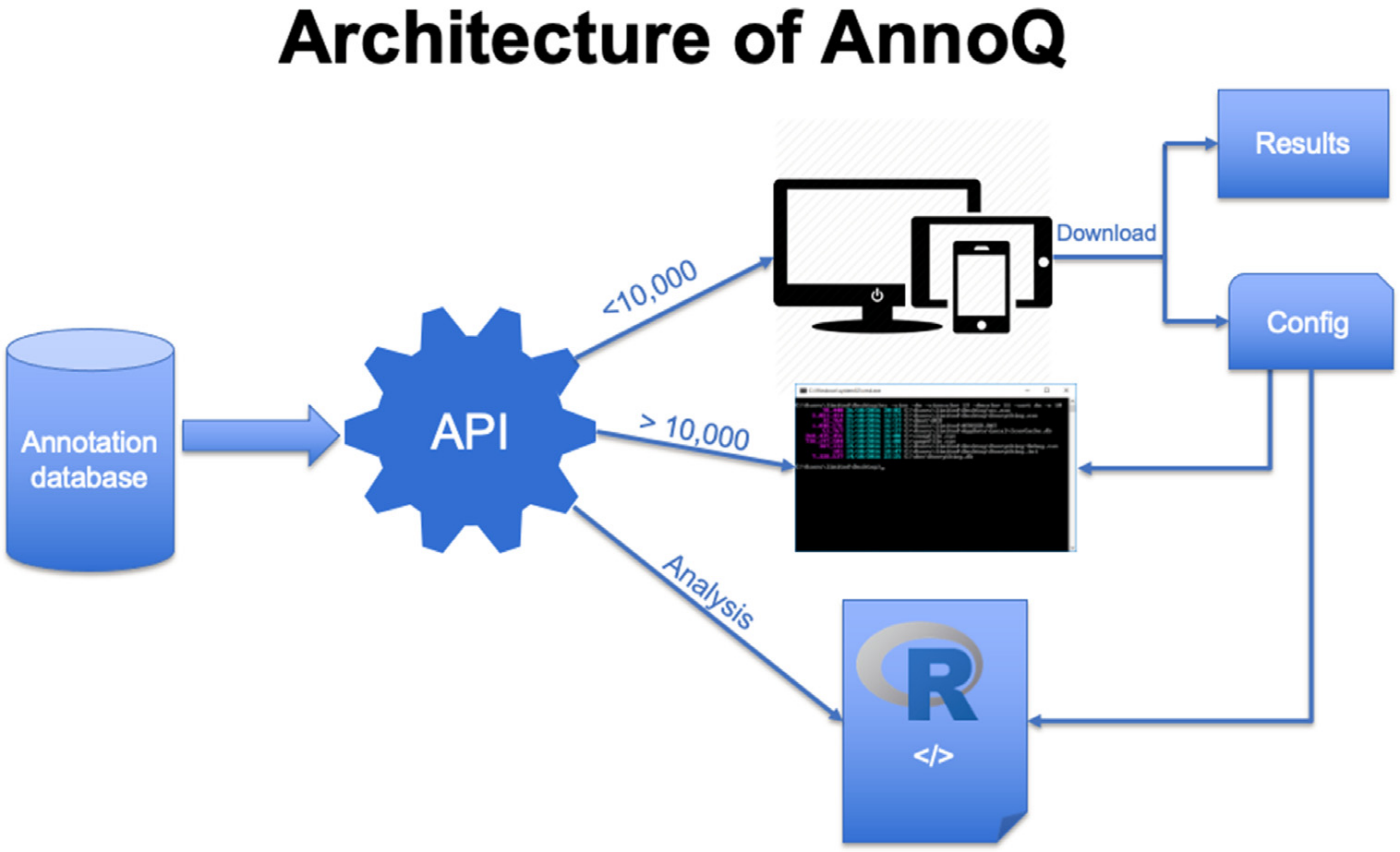
Architecture of AnnoQ platform. The Annotation Data (green box) are gathered from WGSA, PANTHER and PEREGRINE, and are used to annotate the variant dataset (yellow box) to create the annotation database. Users can query the annotation data through an API. There are three ways to query the data, an interactive web interface, command-line scripts, and a programming package.

The AnnoQ System is composed of three main parts (Figure 2). The first is a comprehensive annotation database (AnnoQ Database) built using the Elasticsearch framework, which supports an interactive web interface and API access. It serves as the backend of the system. The second is an API (AnnoQ API) that supports queries to the database. The third is an interactive web interface (AnnoQ Query UI) that allows users to quickly query the database and annotate their variants. The advantage of this architecture is to provide a centralized annotation platform for users to access annotations from multiple tools and resources without the need to set up or install them. Because all the annotations are pre-generated, the variant annotation can be done by real-time searches through simple queries.

### Annotation database

The backend of AnnoQ is supported by an annotation database, which contains pre-annotated human genetic variants. The annotation data are from three main sources: WGSA (18), PANTHER Functional Annotation (19,20), and PEREGRINE enhancer-to-gene links (25) (Table 1). Currently, the system only supports the annotation of human variants.

**Table 1.** Annotation data types

Annotation data	Original annotation source	Annotation tools
Gene-model based annotation (sequence features)	ANNOVAR, SnpEFF, VEP	WGSA
Epigenomics	ENCODE, FANTOMS, Esembl	
Gene-centric annotation	REVEL, SIFT, FATJMM, Plyphen, etc.	
Allele frequency	1000 Genome, gnomAD, ExAC, etc.	
Disease-related variants	COSMIC, ClinVar, GRASP	
Non-coding SNVs	CADD, funseq2, RegulomeDB	
Functional annotations	Gene Ontology, Reactome Pathways, PANTHER Pathways	PANTHER Service
Enhancer to gene links	PEREGRINE	PEREGRINE

**Figure 3.**
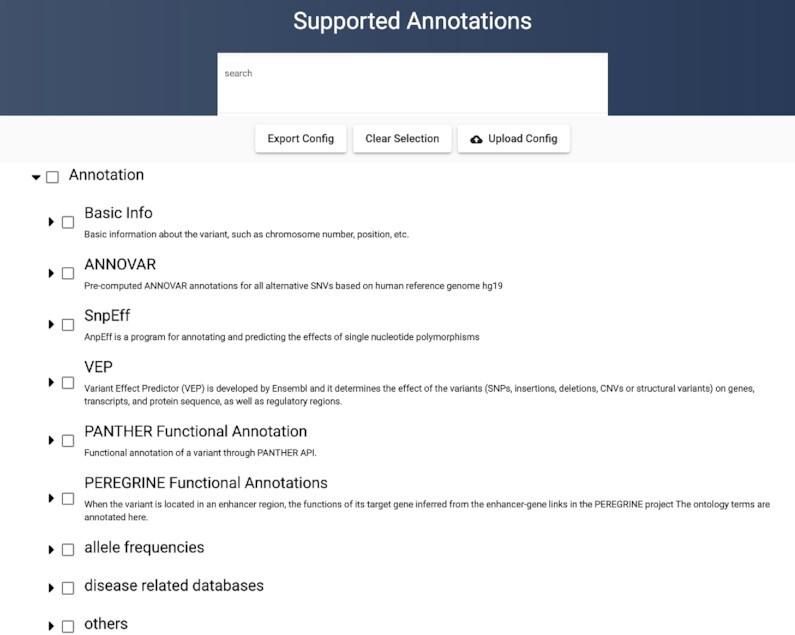
Supported Annotations. The annotations are grouped according to the type of annotation and the source of data. Basic Info is gathered from the genetic variant dataset. PANTHER Functional Annotations are functional annotations from the PANTHER System. PEREGRINE Functional Annotations are annotations to enhancers using the PEREGRINE database. The remaining are from WGSA. PANTHER also provides functional annotations to genes under ANNOVAR, SnpEff and VEP.

There are a total of 607 possible annotation types. The entire list of annotations can be found on our website at http://annoq.org/detail. The annotations are grouped into eight different categories, plus the basic information (Figure 3). The number of annotation types under each group is summarized in Table 2.

**Table 2.** Number of annotation types in each annotation tool or resource group

Annotation tools and resources	Number of annotation types
ANNOVAR	56
SnpEff	79
VEP	75
PANTHER Functional Annotation	48
PEREGRINE Functional Annotation	18
allele frequencies	175
disease related databases	21
others	130

**Figure 4.**
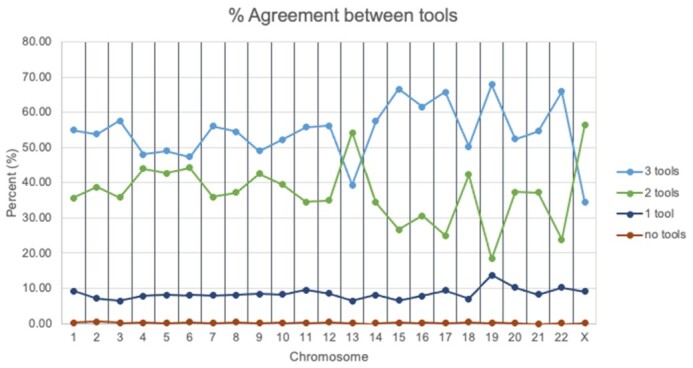
Comparison of gene annotations among different tools. For most variants, all three tools (ANNOVAR, SnpEff and VEP) are in agreement about the type of genomic region the variant appears in; however, a large fraction of variants have some disagreement among tools, and about 10% have different calls from all three tools.

To learn how consistent the annotations are from each of the gene model annotation tools (ANNOVAR, SnpEff and VEP), the percent agreement was calculated between gene lists annotated by each tool and the combined list (see Methods and Materials). For most chromosomes, most variants are given the same annotation by all three tools, except for chromosomes 13 and X (Figure 4, Supplementary Table S1). However, the perfect agreement between tools never passed 70% of variants on a given chromosome (chromosome 19). Furthermore, for the entire human genome, 53.41% of variants have agreement from all three tools (‘3 tools agree’). 38.17% of variants have agreement from two tools, meaning that the annotations from the 3rd tool are a subset of those from the other two (‘2 tools agree’). 8.17% of variants have annotations from one tool as a superset of those from the other two tools (‘1 tool agrees’). Only 0.23% of variants have annotations from at least two tools partially or not overlapping with each other (‘no tool agrees’). These results suggest that users may get different results in their analysis with different annotation tools.

### Annotating variants in non-coding regions

Since the majority of the variants are located in the non-coding regions of the genome, understanding the functional relevance of these variants will have a huge impact on our understanding of the experimental data. It is widely believed that many of these non-coding regions of the genome play pivotal roles in regulating the expression of genes and gene products. In this case, we can annotate a potential regulatory region with the function of the gene it is known (or presumed) to regulate. Non-coding variants are routinely annotated with function based on their proximity to a gene. AnnoQ incorporates these annotations from the PANTHER pipeline. However, some regulatory variants may reside in intergenic enhancer regions, which play a crucial role in regulating the gene expression from a distance. PEREGRINE database has compiled the enhancer-to-gene regulatory links at the genome-wide level (25). AnnoQ uses these data to map variants to enhancer regions and annotate them based on the target genes of these enhancers. As a result, over 2.7 million variants (∼7%) received additional functional annotations in the AnnoQ annotation database.

### Configuration file

**Figure 5.**
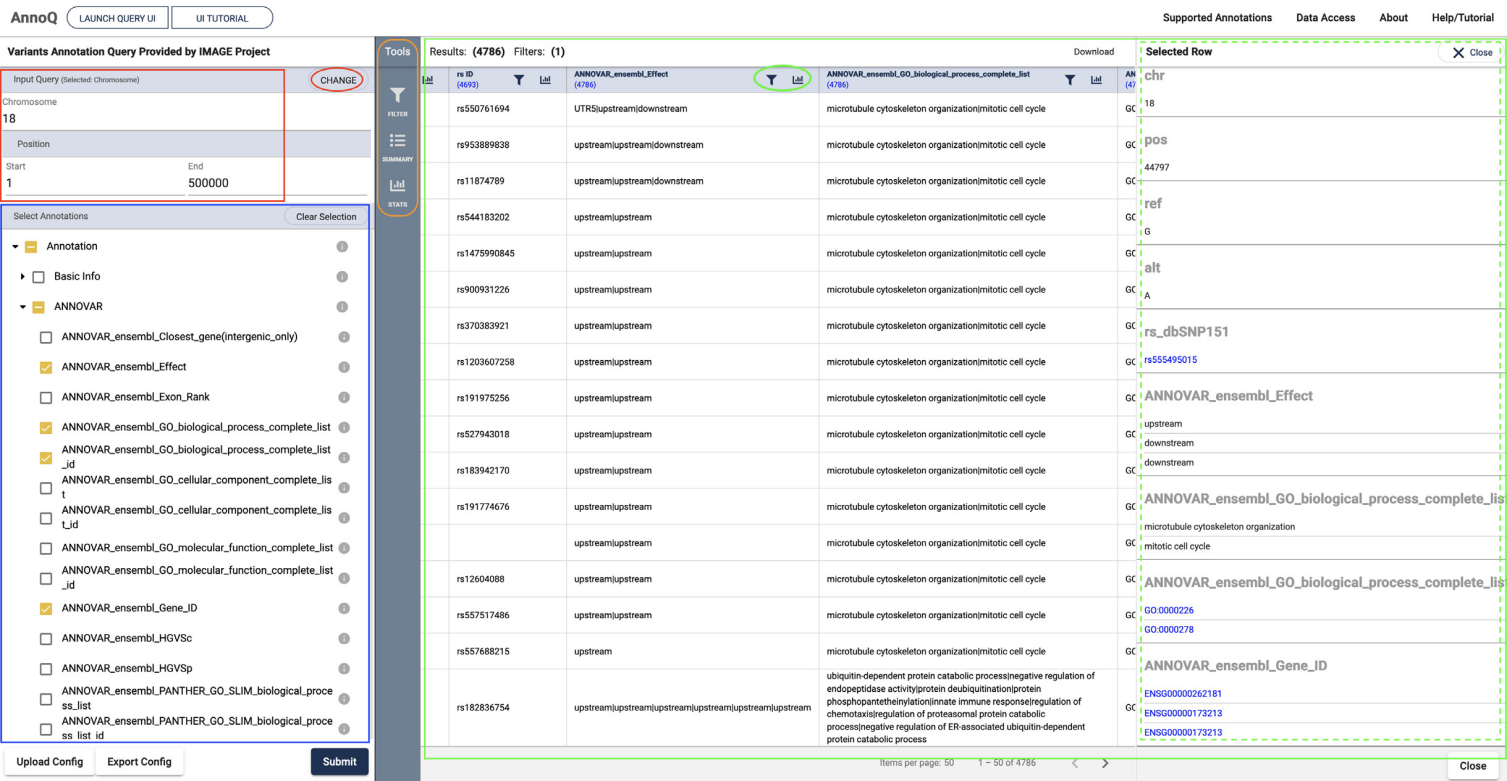
AnnoQ Query UI.

Although AnnoQ includes a comprehensive list of annotation data types, a user rarely needs all of them. For users to reproducibly retrieve a subset of annotation data types, a configuration file mechanism was implemented. The user can go to the interactive AnnoQ website (http://annoq.org/search) (Figure 5) or the Supported Annotation page (http://annoq.org/detail) (Figure 3), browse the annotation tree, select the ones that are relevant to their analysis, and then click the Export Config button. The configuration file will be saved on the user's computer and can later be used by uploading to the interactive webpage or be embedded in the programmatic queries using API.

One major advantage of using the configuration file is that it allows users to perform analyses using a consistent set of annotation data throughout the entire period of the project. This implementation also allows users to create multiple configuration files to test and share them with collaborators.

### Interactive web interface

The interactive web interface (AnnoQ Query UI) (Figure 5) can be launched by clicking the ‘LAUNCH QUERY UI’ button from the home page or directly at http://annoq.org/search. This interface serves the following purposes. First, users can annotate a list of variants and download the results. Second, users can perform gene ID or full-text searches to retrieve lists of variants annotated to the search terms. Third, users can create configuration files based on the results for future queries. Below is a brief description of how to use the UI. A detailed tutorial is available online at https://uscbiostats.github.io/annoq-site/docs/tutorials/ui-query.

### Input search terms

The interactive interface has two panels. The left panel allows users to input the search terms (red box, Figure 5) and select annotation types (blue box, Figure 5). The right panel displays the results (green box, Figure 5). The search terms can be entered in the ‘Input Query’ box on the left panel. There are five supported search types described below that can be selected by clicking the ‘CHANGE’ button (red circle, Figure 5).

1. **Chromosome location**. Users can specify the chromosome number and the start/end positions. AnnoQ will return all variants in the specified region with the user's selected annotations.
2. **VCF file**. Users can upload a list of variants (<10,000) in VCF format (Variant Call Format, https://samtools.github.io/hts-specs/VCFv4.3.pdf). A sample data VCF file is available by clicking the ‘Sample VCF File’ button. AnnoQ will return the annotations to the variants in the file.
3. **Gene product**. Users can input a UniProt (26) ID (e.g. ZMYND11) to retrieve all the variants located within the gene region. AnnoQ will return the selected annotations to these variants.
4. **rsID**. Users can input a dbSNP (27) rsID (e.g. rs559687999) to retrieve the annotations to it.
5. **Keyword Search**. This function is particularly useful. Users can input a keyword in free-text (e.g. Signaling by GPCR), and AnnoQ will return all the variants annotated to the term by any annotation tools.

### Select annotation data

The annotation data types are organized in a tree-style structure and are available on the lower part of the left panel (blue box, Figure 5). An information icon to the right of each annotation term takes the user to a page with detailed descriptions and references. One can click the check box of a group term, e.g. ANNOVAR, to select all annotations to the tool, or expand to the tree and select individual annotation data types. At least one annotation data type needs to be selected to get the results. If a configuration file is available, it can be uploaded using the Upload Config button at the bottom of the panel.

### Retrieving results

The results are displayed on the right panel of the page (green box, Figure 5). Each row is for one variant. The first 5 columns are basic information, which includes chromosome number, position, reference base, the alternative allele, and dbSNP rs ID, followed by the selected annotation data in the remaining columns. In most cases, not all columns have annotation data, and they are shown as blank. To view the annotation data more easily, users can click on any row to have a pop-up table (dashed green box, Figure 5) that includes all the annotations to the variant. If a variant is mapped to a dbSNP rsID, or annotation is a GO or Ensembl Gene ID, it includes a hyperlink to link to the respective source webpage.

In order to help users to navigate the results, tools have been built to filter the results and view the summary statistics (orange box, Figure 5). Users can simply click an icon to expand the panel, and narrow down the results by using the filters or summary statistics. Users can also click the icon in the column header (green circle, Figure 5) to filter the results or view the statistics of the data in the respective column. A detailed description of using filters is described in Supplementary Figure S1 of the Supplemental Materials.

The annotation results can be downloaded by clicking on the Download button.

A few use cases and sample workflows that are unique to, but commonly used in AnnoQ are illustrated in Supplementary Figure S2 of the Supplemental Materials.

### Data access API

Besides the interactive web interface, AnnoQ API also provides other ways to access the annotation data (Figure 1). For users who want to annotate a large number of variants or multiple lists of variants, they can do so by accessing the API directly using a command-line script or a programming package. AnnoQ API provides powerful search capability with great flexibility. Although the interactive web interface can handle a variant list of any size and is highly useful for browsing the data and query annotations, it is recommended to annotate large numbers of variant data, especially for large association studies, by sending queries through our API directly. Multiple tools, such as the ‘curl’ command in Linux, can help users to perform these queries. The details about how to use API can be found at https://uscbiostats.github.io/annoq-site/docs/tutorials/api.

The AnnoQ API also allows users to access the data with various programming languages. This is particularly useful when users want to retrieve annotations on the fly while analyzing the data. To facilitate the users who use R to analyze data, an R package called AnnoQR has been developed. The details about how to use AnnoQR can be found at https://uscbiostats.github.io/annoq-site/docs/tutorials/r-package. We are working on packages for other programming languages and will add them to our repository soon.

## DISCUSSION

Annotation Query (AnnoQ) is designed for quick and easy annotation of genetic variants. It annotates the variants with a comprehensive list of annotation data types in advance, supported by a sophisticated search engine and search API. Users can annotate genetic variants by simply querying the database without installing any tools. Although some features are present in other similar tools, e.g. dbNSFP (28), wANNOVAR (29) or MyVariant.info (30), AnnoQ provides a more comprehensive set of features to support a diverse user community. Table 3 provides a comparison of some key features between AnnoQ and other annotation tools.

**Table 3.** Comparison of features between AnnoQ and other annotation tools

	Tools					
	AnnoQ	ANNOVAR	SnpEff	VEP	dbNSFP	MyVariant.info
Require download and installation	No	Yes	Yes	Yes	Yes	No
Pre-annotated	Yes	No	No	No	Yes	Yes
Annotations from Multiple Tool	Yes	No	No	No	Yes	No
Realtime response	Yes	No	No	No	No	Yes
Complex Query functions	Yes	No	No	No	No	Yes
Interactive User Interface	Yes	Yes*	No	No	No	No
Functional annotation	Genes, GO and pathways	Genes only	Genes only	Genes only	Genes only	Genes only

### An interactive query tool with visualization

One of the highlights of the AnnoQ System is its interactive web interface. Instead of routine annotation tools requiring users to install and run them, AnnoQ pre-annotates a large collection of variants and serves as a query tool. The sophisticated search framework and API make it easy for users to work with large volumes of data and are suitable for researchers with different computational and bioinformatics backgrounds. The simple user interface allows users to perform quick queries or annotate a small variant dataset. The results are presented in multiple forms, which makes it easy for users to evaluate them and make adjustments in annotation data type selection.

### Up-to-date functional annotations

It is essential to provide the most up-to-date functional annotations for accurate analysis and interpretation of the data (31). AnnoQ obtains function annotations directly from the PANTHER Classification System (19) API, ensuring that it provides users with up-to-date functional annotations, including annotations from GO, PANTHER, and Reactome pathways. In addition, the system is also linked to PEREGRINE (25), a genome-wide enhancer-gene link database, to provide functional annotations to non-coding variants.

### Comprehensive and consistent in annotation

As we show here, genomic region annotations from different annotation tools are slightly different (Figure 4 and Supplement Table S1). It's been shown that such differences may significantly impact the variant annotation, and thus the data analysis (16). AnnoQ aggregates annotations from multiple tools under one platform. This becomes helpful because users have options to select a more comprehensive annotation dataset by querying and mix-and-matching annotations from multiple tools. Again, this can be done through simple queries with real-time results.

With such abilities to select annotation data types, it is necessary to keep track of the selection to ensure the consistency of the annotation. The configuration file in AnnoQ serves this purpose. Once the annotation data types are selected, users can export the selection to a configuration file, which can be uploaded later to ensure that all analyses are done with the same annotation data type. The file can also be shared among collaborators for consistency.

### Search capability

Although the main purpose of AnnoQ is to annotate genetic variants, it can also serve as a variant search tool. The data structure is built such that it is also capable of supporting more general query functions, such as free-text keyword search. For example, users can input a gene (UniProt ID) and retrieve all variants located in the gene region. Users can also conduct a free-text keyword search, for example, a pathway name, and retrieve all variants annotated to the pathway.

The other feature of the AnnoQ search is its extensibility. The infrastructure is based on the Elasticsearch framework and has hundreds of millions of entities/docs but still achieves real-time query capability. It is horizontally scalable, meaning that the data can be expanded by adding more computer nodes without affecting the configuration and performance of the existing ones. This is extremely useful because we plan to add variants from the entire dbSNP, which contains more than 20 times more variants than the current system. We do not expect any effect on performance with such an increase in data size.

### Software packages for programmatic access to annotations

In addition to the interactive web interface for variant annotation queries, the API design also allows access to the annotation data programmatically. Software packages can be developed to allow users to embed annotation functionalities in their scripts and retrieve annotations while analyzing the data. AnnoQR, an R package, was developed for this purpose. This is particularly useful for researchers who are more skilled in programming to annotate large variant datasets.

In conclusion, we have developed Annotation Query (AnnoQ), an integrated platform with an easy-to-use user interface for genetic variant annotation. AnnoQ provides the most up-to-date functional annotations. Its sophisticated API design and powerful search capability allow users to not only quickly annotate variants, but also perform complex search and analysis of the annotation data. The system will support researchers with a wide range of backgrounds and research interests, but more importantly, it will certainly benefit researchers with less computation and bioinformatics skills, such as bench scientists and statisticians.

### Future development

We have shown that AnnoQ is a useful platform for the annotation of genetic variants, and it can serve researchers with diverse backgrounds. Nevertheless, there are still rooms to improve. Here are a few areas that we plan to expand and improve in the future.

1. *Improve the coverage of human variants*. Currently, AnnoQ contains ∼39 million pre-annotated variants from HRC. We plan to cover the entire dbSNP in the near future.
2. *Expand to cover additional genomes*. Some of the tools in AnnoQ, such as ANNOVAR and PANTHER, support annotation of other genomes (e.g. mouse, fly). We plan to support these genomes in AnnoQ in the future.
3. *Build additional visualization tools to help the interpretation of the results*. The results from AnnoQ sometimes can be overwhelming. A few visualization tools and summary statistics have been built to help the user to view the results (Supplementary Figure S1 in the Supplemental Materials). Additional tools will be developed, especially by incorporating results from the tool comparison analysis (Figure 4) to help users to make better decisions in selecting the annotation types, and interpreting the results.
4. *Include additional annotations*. We plan to add more annotations to the AnnoQ system in the future, including the microbiome, metabolomic annotation data, and more pathways from the PanthwayCommons effort.

## DATA AVAILABILITY

All data described in this manuscript are freely available through API download. All programming codes are available at GitHub repositories described in the manuscript.

## Supplementary Material

gkac418_Supplemental_File
