# Supplementary material for: Annotation Query (AnnoQ): an integrated and interactive platform for large-scale genetic variant annotation
Source: Nucleic Acids Res. 2022 May 30;50(W1):W57–65. doi: 10.1093/nar/gkac418 (PMC9252745; doi:10.1093/nar/gkac418)

# Supplemental materials

**Table S1. Comparison of gene annotations by three gene model-based tools.**


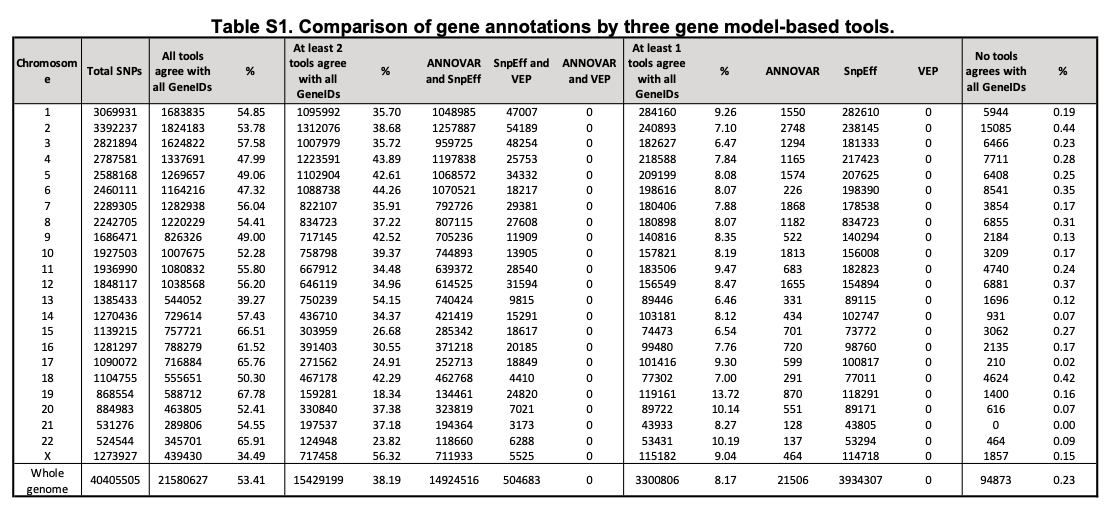


This table presents detailed comparison data from each individual chromosome. The method was described in the Materials and Methods section. Here is a brief description about the data in each column.

*Column A.* The chromosome number. Row 26 is the results from the entire genome.

*Column B.* Total number of variants in the respective chromosome.

*Column C.* The number of variants that have a perfect match of gene annotations from all three tools.

*Column D*. Percent of Col C over Col B.

*Column E.* The number of variants that have genes annotated by any two tools matching those in the combined list.

*Column F*. Percent of Col E over Col B.

*Columns G-I*. The number of variants that have genes annotated by the two tools in row 2 matching those in the combined list.

*Column J*. The number of variants that have genes annotated by any one tool matching with those in the combined list.

*Column K*. Percent of Col J over Col B.

*Columns L-N*. The number of variants that have genes annotated by the tool in row 2 matching those in the combined list.

*Column O*. The number of variants that no tool provides matching results as those in the combined list.

*Column P*. Percent of Col O over Col B.

The table shows the following main results.

- - - 1. All three tools provide identical annotations between 34.49% (Chr X) and nearly 68% (Chr 19) variants in each individual chromosome, with an average of 53.41% average for the entire genome.
      2. When there are only two tools agreeing on each other and matching the combined list, they are mostly from ANNOVAR and SnpEff, meaning VEP annotates fewer genes in these variants. Occasionally, they are from SnpEff and VEP, suggesting that ANNOVAR annotates fewer genes in this case. They are never from ANNOVAR and VEP, suggesting that SnpEff never annotates fewer genes than the other two in this category.
      3. When there is only one tool matching the combined list, most of them are from SnpEff. Only a few are from ANNOVAR when SnpEff annotates fewer genes than ANNOVAR.
      4. There are very few variants (0.23%) that have three tools not agreeing with each other.

These results show that SnpEff annotates the most genes. Even so, there are a fraction of variants that have more genes annotated by ANNOVAR. VEP annotates to the least number of genes. Depending on which annotation tool is used, the annotation results for the variants can differ drastically. Therefore, it is important for users to know such differences, and make the right decision in selecting the annotation data.

Figure S1. Filtering tools and summary statistics of the AnnoQ results. There are three tools to help users to visualize the annotation results and view statistics. **A**. Filter the results by clicking the Filter icon. On the filter page, the user can click the filter box, and a list of annotations will appear in the drop-down menu. Users can also start to type the annotation term, and the box will be automatically filled. Users can select any annotation. The results page will refresh to only display rows that have annotation data in the filtered annotation term. Multiple terms can be entered to show results that have annotations in all the filtered terms. **B**. View the search summary by clicking the Summary icon. On the Search Summary page, a list of selected annotations and numbers of rows that have the annotations are displayed. A filter icon and a statistics icon are displayed for each annotation also. A user can click those icons to filter the results page or to see the annotation statistics. **C**. View annotation statistics by clicking the Stats icon. The user can view the statistics of a particular annotation type by selecting it from the drop-down menu at the top of the page. There are three different graphs available for each type. The first is a pie chart to show the fraction of results that have the annotation to this particular annotation type. The second is a bar chart to show the number of variants annotated to a particular term. The exact number can be seen when mouse-over the bar. Both of the above graphs are under the General tab of the page. The third graph is under the Other tab, and it shows a plot of the number of annotations over the chromosome coordinates.

Please note that the search summary and stats are only for the current results. For example, if a user uses a filter to narrow down the results, the summary and stats are for the filtered results. The user can go to the Filter page and remove the filters in order to view all the data.

**
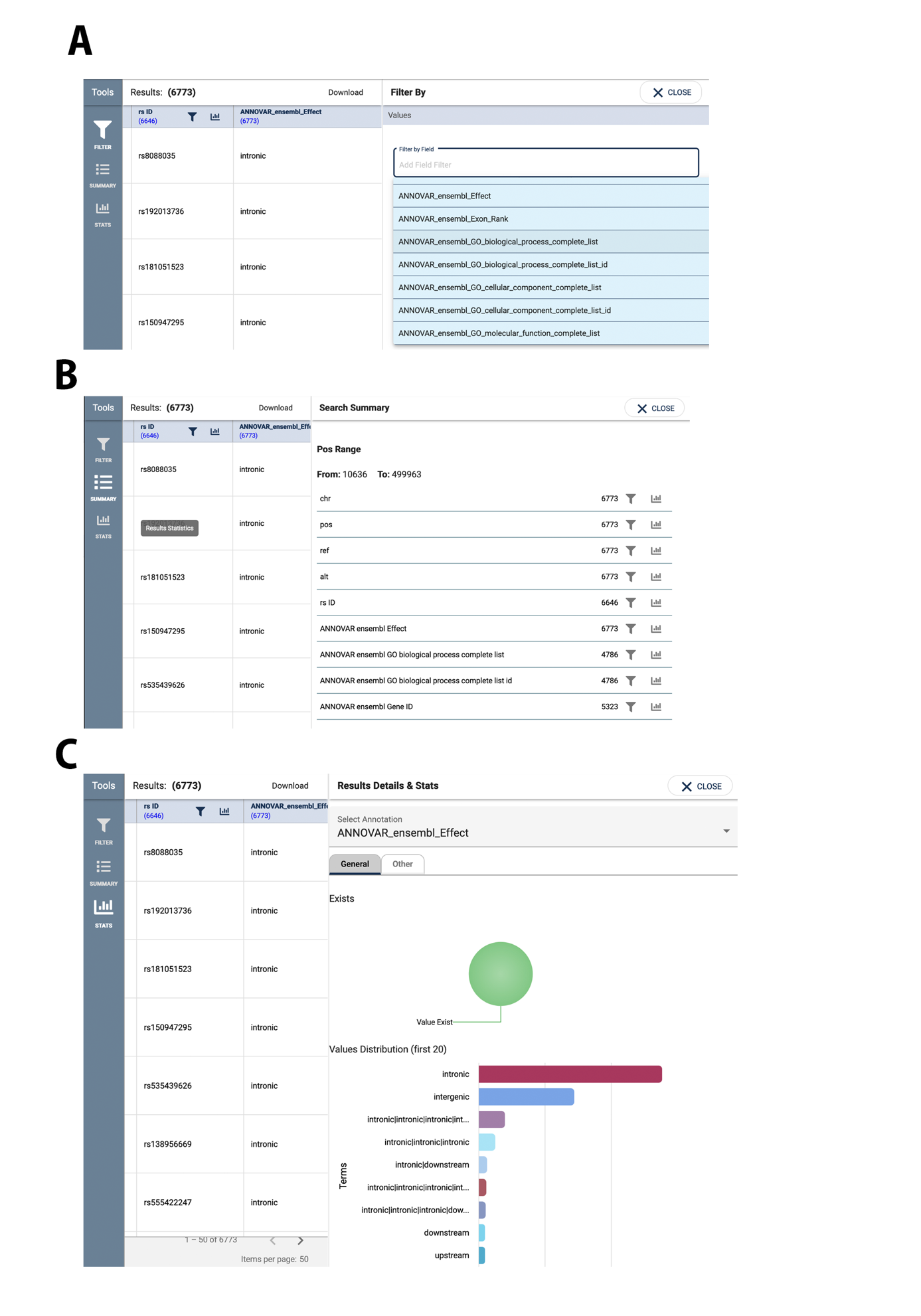
**

Figure S2 Sample use cases and workflows of AnnoQ.

This figure outlines three sample use cases. **A.** Annotation of the large genetic variant list in VCF files. This is the most commonly used workflow. The user first inputs a shortlist of variants, also in the VCF file, through the AnnoQ interactive query interface, and then selects annotation types. The purpose of this is to select the desired annotations based on the results. Once the user is satisfied with the choice of the annotations, they can be exported to a configuration file. Then the user can use the command line to query large variant lists or even multiple lists through AnnoQ API with defined annotation types using the configuration file. The advantage of this workflow is that the user can annotate additional lists in the future with exactly the same annotations. The same configuration file can also be used in AnnoQR. **B.** Query for variants in the enhancer regions that regulate genes involved in a particular biological process or pathway. The user first selects “Keyword Search” as the search type, and then enters the keyword in free-text. It can be a name of a process or a pathway. “PEREGRINE Functional Annotation” should be selected as the annotation type. The results will contain both the genes that are annotated to the GO annotations or pathways with the keyword and all the variants in the enhancer regions regulating these genes predicted by PEREGRINE. **C.** Query for variants related to a particular gene of interest. The user can select “Gene Product” as the search type and enter the UniProt ID in the search box. The user can select ANNOVAR, SnpEff, or VEP to retrieve all variants associated to the gene annotated by these tools. The user can also select PEREGRINE to retrieve variants located in the enhancer regions regulating the gene.


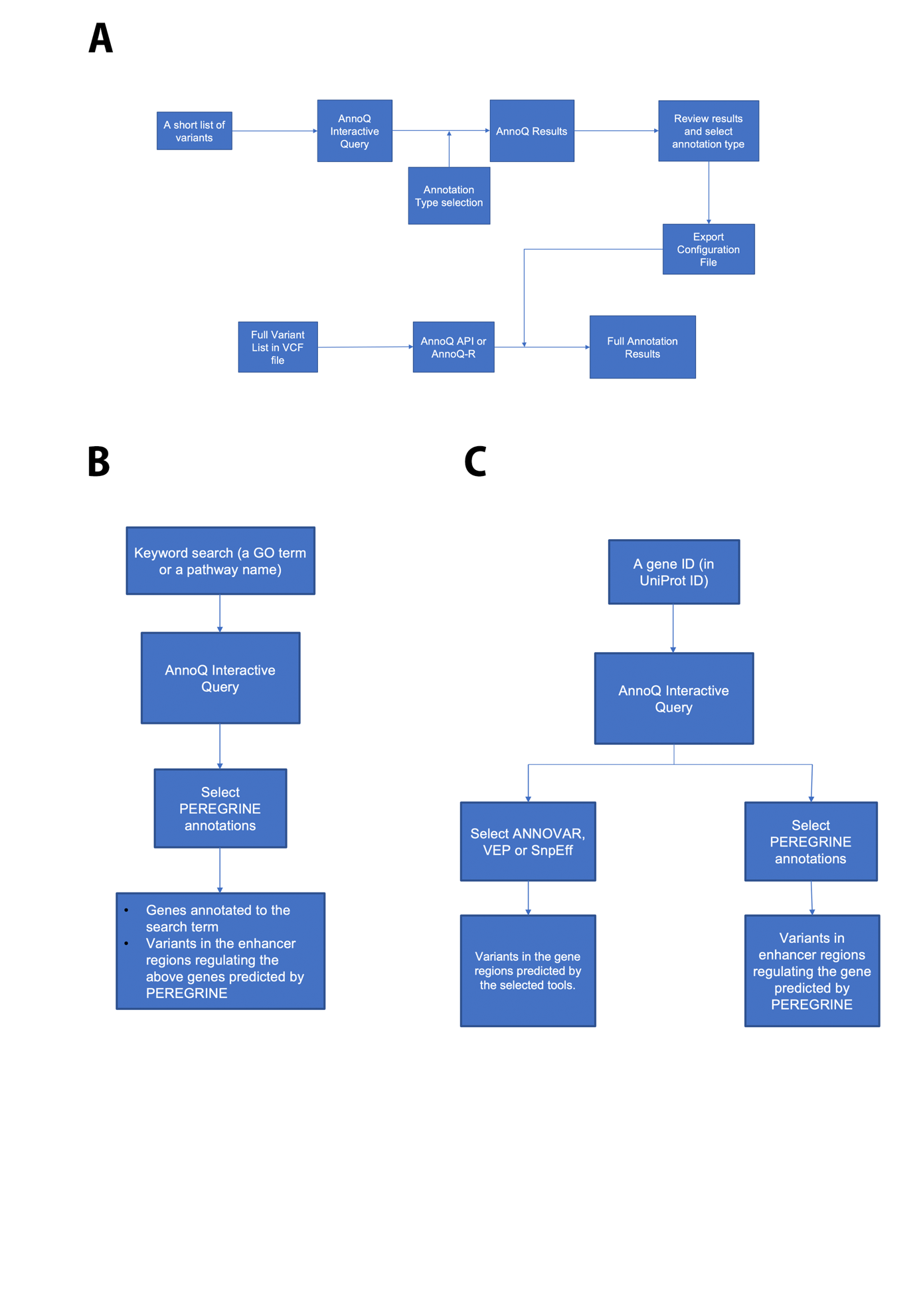

Supplement: gkac418_Supplemental_File [file gkac418_supplemental_file.docx]
